# MUC3A induces PD-L1 and reduces tyrosine kinase inhibitors effects in EGFR-mutant non-small cell lung cancer

**DOI:** 10.7150/ijbs.57964

**Published:** 2021-04-12

**Authors:** Yuan Luo, Shijing Ma, Yingming Sun, Shan Peng, Zihang Zeng, Linzhi Han, Shuying Li, Wenjie Sun, Jieyu Xu, Xiaoli Tian, Feng Wang, Qiuji Wu, Yu Xiao, Junhong Zhang, Yan Gong, Conghua Xie

**Affiliations:** 1 Department of Radiation and Medical Oncology, Zhongnan Hospital of Wuhan University, Wuhan, China.; 2 Department of Geriatrics, Renmin Hospital of Wuhan University, Wuhan, China.; 3 Department of Biological Repositories, Zhongnan Hospital of Wuhan University, Wuhan, China.; 4 Hubei Key Laboratory of Tumor Biological Behaviors, Zhongnan Hospital of Wuhan University, Wuhan, China.; 5 Hubei Cancer Clinical Study Center, Zhongnan Hospital of Wuhan University, Wuhan, China.

**Keywords:** MUC3A, non-small cell lung cancer, EGFR, PD-L1.

## Abstract

The immune checkpoint ligand programmed death-ligand 1 (PD-L1) and the transmembrane mucin (MUC) 3A are upregulated in non-small cell lung cancer (NSCLC), contributing to the aggressive pathogenesis and poor prognosis. Here, we report that knocking down the oncogenic MUC3A suppresses the PD-L1 expression in NSCLC cells. MUC3A is a potent regulator of epidermal growth factor receptor (EGFR) stability, and MUC3A deficiency downregulates the activation of the PI3K/Akt and MAPK pathways, which subsequently reduces the expression of PD-L1. Furthermore, knockdown of MUC3A and tyrosine kinase inhibitors (TKIs) in EGFR-mutant NSCLC cells play a synergistic effect on inhibited proliferation and promoted apoptosis *in vitro*. In the BALB/c nude mice xenograft model, MUC3A deficiency enhances EGFR-mutated NSCLC sensitivity to TKIs. Our study shows that transmembrane mucin MUC3A induces PD-L1, thereby promoting immune escape in NSCLC, while downregulation of MUC3A enhances TKIs effects in EGFR-mutant NSCLC. These findings offer insights into the design of novel combination treatment for NSCLC.

## Introduction

Lung cancer remains the leading cause of cancer-related mortality worldwide. Non-small cell lung cancer (NSCLC) accounts for approximately 85% lung cancer cases. Therapeutic antibodies blocking programmed death-ligand 1 (PD-L1) brought striking regression of NSCLC. However, only 20% patients responded to the anti-PD-L1 therapy 1. The expression levels of PD-L1 on the surface of tumor cells were positively correlated with the induction of the inflammatory cytokine interferon-γ 2. Previous studies suggested that stable expression of mutated epidermal growth factor receptor (EGFR) in immortalized bronchial epithelial cells promoted PD-L1 expression, and that EGFR tyrosine kinase inhibitors (TKIs) inhibited PD-L1 expression in EGFR-mutant NSCLC cells 3. In addition, epidermal growth factor (EGF) was reported to induce PD-L1 expression through the IL‐6/JAK/STAT3 signaling pathway in EGFR-mutant NSCLC cells 4.

The mucin (MUC) family proteins contain tandem repeat structures, in which the proportions of proline, threonine, and serine are high. The human MUC family consists of members from MUC1 to MUC21, which are sub-classified into the secreted (such as MUC2, MUC5AC, MUC5B, and MUC6) and transmembrane forms (such as MUC1, MUC4, MUC3, and MUC16) 5. MUCs are large glycoproteins and actively participate in tumor proliferation and metastasis 6. MUC1 was reported to be upregulated in triple negative breast cancers (TNBCs) 7. It induced PD-L1 transcription through MYC and NF-κB/p65 pathways in basal B TNBC cells. Targeting MUC1 C-terminal with genetic and pharmacologic approaches suppressed PD-L1 expression 8. Moreover, MUC4 was highly glycosylated, and the expression of EGFR was modulated by MUC4 9.

MUC3A is also highly glycosylated. It contains a sperm protein, enterokinase, agrin (SEA) domain, and an EGF domain in its extracellular segment 10. MUC3A is highly expressed in NSCLC cells and rarely expressed in normal pulmonary epithelial cells, making it a promising tumor biomarker for lung cancer 11. In gastric, pancreatic, breast, colorectal, renal, and prostate cancers, high expression of MUC3A is an independent factor for poor prognosis 12-17.

MUC family members regulate PD-L1 expression 18, which is associated with the prognosis of immunotherapy for lung cancer. MUC proteins also modulate EGFR 19, whose mutation is popular in NSCLC. EGFR was reported to modulate PD-L1 expression via the PI3K/Akt and MAPK pathway 20. Therefore, we speculate that MUC3A, containing the EGF domain, may induce PD-L1 expression through PI3K/Akt and MAPK pathway. In the present study, we demonstrated that MUC3A could increase EGFR stability and reduce the effects of TKIs on NSCLC cells. MUC3A was positively correlated with PD-L1 in NSCLC, and the increased MUC3A and PD-L1 levels both indicated poor prognosis of NSCLC patients. Knockdown of MUC3A decreased EGF-induced PD-L1 in EGFR-mutated NSCLC via blocking PI3K/Akt and MAPK pathways. MUC3A deficiency also enhanced TKIs-induced proliferation inhibition and apoptosis promotion in NSCLC cells *in vitro* and *in vivo*. Our studies indicated that MUC3A might be a potential target in lung cancer treatment strategies.

## Materials and Methods

### Tissue microarray and bioinformatics analysis

The NSCLC tissue microarray was purchased from Outdo (Shanghai, China), including 92 lung adenocarcinoma (LUAD) and their paired para-carcinoma tissues. The samples come from National Human Genetic Resources Sharing Service Platform (2005DKA21300). Both the intensity and positive percentages of immunohistochemistry (IHC) were used to examine the MUC3A and PD-L1 expression: the IHC H-score (values 0-400) = the scores for intensity of positive staining (less than 5% scored “0”; 5-24% scored “1”; 25-49% scored “2”; 50-74% scored “3”; and more than 74% scored “4”) × the percentage of positive-stained cells × 100 21. In the cancer tissues of all the 92 cases, the median MUC3A H-score was 140.

### Animals

Five-week-old female BALB/c nude mice were purchased from Charles River Laboratory Animal Technology Co., Ltd, Beijing, China. Mice were housed and handled according to the guidelines of Wuhan University Animal Care Facility and National Institutes of Health. The H1975 cells (5 × 10^6^ cells/mouse) were subcutaneously injected into the right armpit. The mice were randomized into 4 groups: control, MUC3A deficiency, AZD-9291 (5 mg/1 kg/day), and MUC3A deficiency with AZD-9291. The sizes of the subcutaneous tumors were recorded every day. Tumor volume (V) was calculated using the formula: V = π/6 × (major axis) × (minor axis)^2^.

### Cells

Human NSCLC cells (H1975, H1299 and PC9), large cell lung cancer cells (H460), lung mucosal epithelial cells (H292), normal lung epithelial cells (BEAS-2B) were purchased from the Type Culture Collection of the Chinese Academy of Sciences, Shanghai, China. Lung cancer cells were maintained in RPMI-1640 medium, and BEAS-2B cells were maintained in DMEM medium. Mediums were supplied with 10% fetal bovine serum, 100 mg/mL streptomycin and 100 units/mL penicillin. Cells were cultured in a 37 °C incubator (Sanyo, Japan) with 5% CO_2_. All cells passed the short tandem repeat (STR) analysis of Guangzhou Cellcook Biotech Co., Ltd, China.

### Cell viability assay

Cells were seeded into 96-well plate and treated with AZD-9291 and Gefitinib (MCE Ltd., China) at different doses for 48 h. Cell viability was detected by the CCK-8 kit (Dojindo Ltd., Japan) according to the manufacturer's instructions. The optical density was measured at 450 nm through a microplate reader (Rayto Ltd., China).

### Flow cytometry

Cells were incubated with Gefitinib (10 μM) and AZD-9291 (0.1 μM) for 48 h. Suspended and adherent cells were both collected. Cell apoptosis was detected by Annexin VFITC/PI Apoptosis Kit (BestBio Ltd., China) according to the manufacturer's instructions and analyzed by flow cytometry (FACS Aria III, BD, USA).

To detect cell membrane expression of PD-L1, cells were digested and incubated in 100 μL PBS containing 2% goat serum at room temperature for 20 min. After washed with PBS for 3 times, cells were incubated with the anti-PD-L1 antibody (listed in Supplemental Table S1) in the dark at 4°C for 30 min. After washing with PBS, the stained cells were resuspended in 400 μL PBS. The samples were then analyzed by flow cytometry (FACS Aria III, BD, USA).

### RNA isolation, RT-PCR and qRT-PCR

TRIzol (Sangon Ltd., China) was used to extract total RNA. RNA concentration was detected by a Nanodrop spectrophotometer (Thermo Scientific Ltd., USA). Total RNA (1 μg) was used for the synthesis of first-strand cDNA and reverse transcription reactions were conducted using HiScript® II Q RT SuperMix for qPCR kit (Vazyme Ltd., China). The following primers were used: PD-L1 (forward 5'- GCTGCACTAATTGTCTATTGGGA -3' and reverse 5'- AATTCGCTTGTAGTCGGCACC -3'); GAPDH (forward 5'- GGAGCGAGATCCCTCCAAAAT -3' and reverse 5'- GGCTGTTGTCATACTTCTCATGG -3'). The qRT-PCR reactions were performed using a CFX96 qRT-PCR system (Applied Biosystems Ltd., USA). We used the 2-ΔΔCT method to calculate the fold changes. Data were normalized to GAPDH levels.

### Immunoblotting

RIPA lysis buffer (Beyotime Ltd., China) containing protease and phosphatase inhibitor mixture (Sigma Chemical Ltd., USA) was used to extract the whole cells lysis on ice for 30 min. The supernatant was collected after the cell lysates centrifugation at 13,000 g for 20 min at 4 °C. The protein concentration was determined by BCA assay (Beyotime Ltd., China). Protein samples were separated by 7.5-12.5% SDS-PAGE, and the proteins were then transferred to polyvinylidene fluoride membranes. Non-fat milk (5%) was used to block non-specific binding sites. Primary and secondary antibodies used for detection were listed in Supplemental Table S1. Then, the specific bands were visualized with an enhanced chemiluminescence kit (Bio-Rad Ltd., USA) and exposed to the ChemiDoc XRS + system (Bio-Rad Ltd., USA). The Image J program was used to quantify the protein levels.

### Histology and IHC

The tumor tissues were fixed with 10% formalin and embedded in paraffin. Tumor tissue sections were used for hematoxylin and eosin (H&E) staining and IHC. IHC was used to detect MUC3A and PD-L1 expression in tumor tissues. Anti-MUC3A antibody and Anti-PD-L1 antibody were listed in Supplemental Table S1.

### Statistical analysis

Each experiment was performed for at least 3 times, and data were presented with a representation of at least 3 individual experiments. A two-tailed Student's t-test and one-way analysis of variance (ANOVA) were used to evaluate the statistical significance of different groups. Data were analysed with GraphPad Prism. *P* values < 0.05 were considered as statistical significance.

## Results

### MUC3A expression was positively correlated with PD-L1 expression in NSCLC

**Figure 1:**
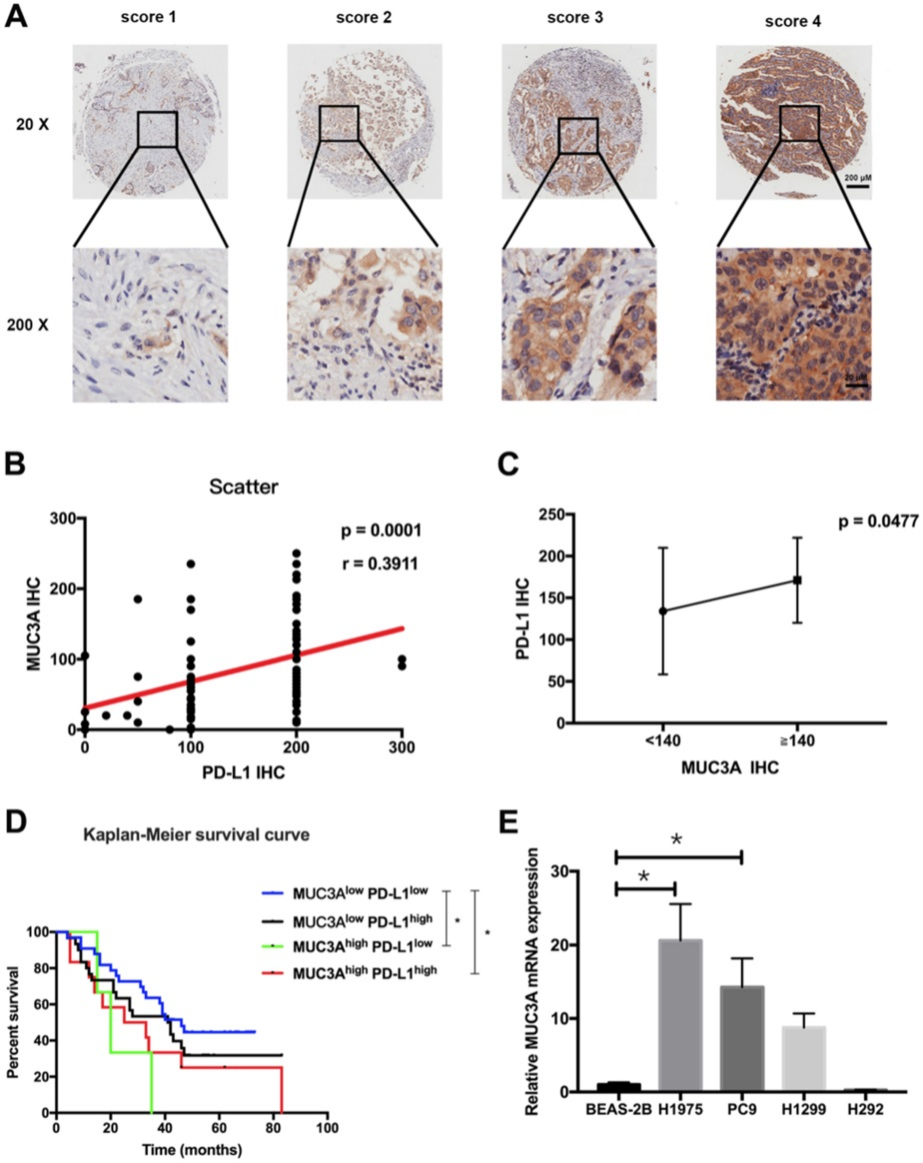
** MUC3A was positively correlated with PD-L1 expression. (A)** Representative IHC images of PD-L1 expression. Scoring was measured by the percentage of positive cells with the following staining intensities: less than 5% scored “0”; 5-24% scored “1”; 25-49% scored “2”; 50-74% scored “3”; and more than 74% scored “4”. **(B)** and **(C)** The level of MUC3A was positively correlated with PD-L1 expression. **(D)** High levels of MUC3A and PD-L1 were related to poor clinical outcomes. **(E)** Relative MUC3A mRNA expression in BEAS-2B, H1975, PC9, H1299 and H292. *, *p* < 0.05.

Tissue microarray containing 92 LUAD and paired para-carcinoma tissues with scoring system (Fig. 1A) was used to confirm the increased MUC3A and PD-L1 levels in LUAD tissues compared with normal lung tissues (Fig. S1). The expression levels of MUC3A and PD-L1 were positively correlated (Fig. 1B). Adenocarcinoma with higher levels of MUC3A (IHC > 140) also had higher expression of PD-L1 than the lower ones (Fig. 1C, *p* < 0.05). Different clinicopathological features of LUAD cases stratified by PD-L1 expression levels were compared, and PD-L1 levels were associated with primary and adjacent carcinoma (*p* < 0.0001, Table S2). Moreover, LUAD patients were sub-grouped into 4 groups by their PD-L1 and MUC3A expression levels, and the increased MUC3A and PD-L1 levels were correlated with increased lymph node metastasis (*p* < 0.05, Table 1). Higher expression of both MUC3A and PD-L1 was associated with poorer prognosis (*p* < 0.05, Kaplan-Meier test, Fig. 1D). To further verify MUC3A expression levels in NSCLC and normal lung epithelial cells, the mRNA levels of MUC3A were measured. MUC3A was highly expressed in LUAD cell lines H1975 and PC9 compared with H1299, and barely expressed in lung mucosal epithelial cells (H292) and normal lung epithelial cells (BEAS-2B) (Fig. 1E). These results suggested that MUC3A expression was upregulated in NSCLC cells and positively correlated with PD-L1 expression.

### Knockdown of MUC3A inhibits EGF-induced PD-L1 expression in EGFR-mutant NSCLC cells

**Figure 2:**
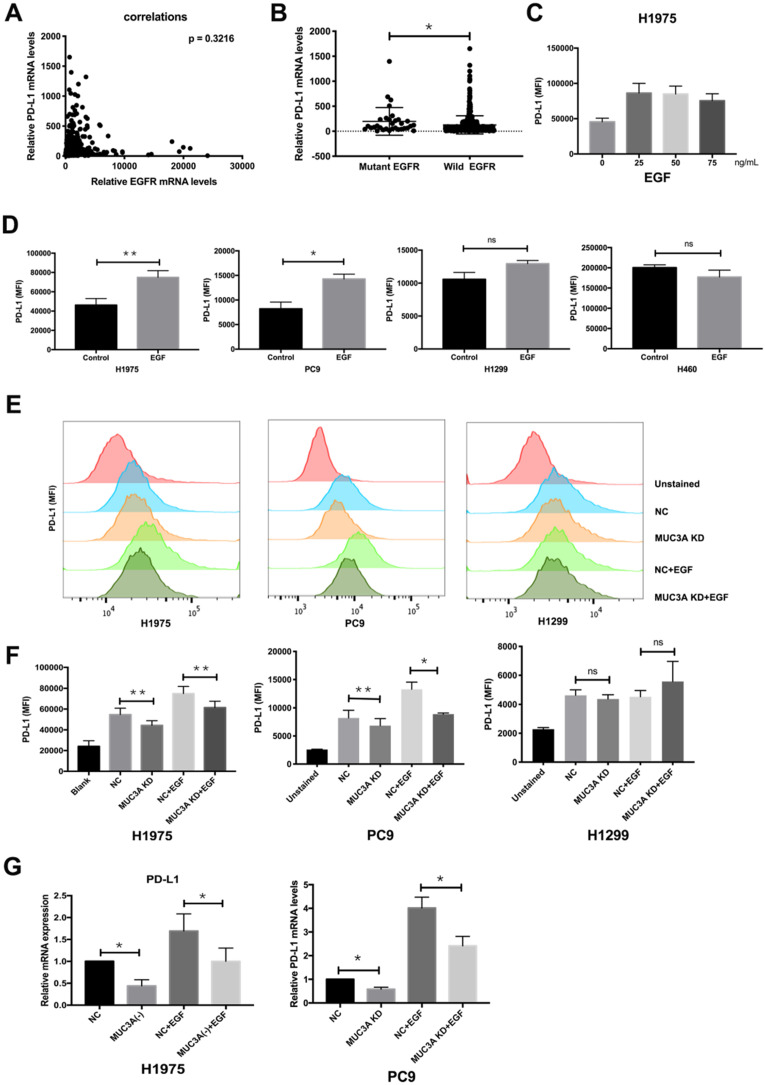
**MUC3A induced PD-L1 in EGFR-mutant NSCLC cell lines. (A)** The mRNA levels of PD-L1 were independent of the mRNA levels of EGFR. **(B)** The mRNA levels of PD-L1 were correlated with EGFR mutation. Data of lung cancer were downloaded from the TCGA database. 544 non-mutation, 32 mutation. **(C, D)** The PD-L1 mRNA levels were determined by real-time PCR analysis after EGF stimulation for 24 hours. **(E, F)** The PD-L1 protein levels were determined by flow cytometry analysis after EGF stimulation for 24 hours. **(G)** The PD-L1 mRNA levels were determined by real-time PCR analysis after EGF stimulation for 24 hours. EGF, 50 ng/mL. *, *p* < 0.05; **, *p* < 0.01; ns: not significant. NC: negative control.

EGF was reported to activate EGFR and then induce PD-L1 expression 22. To investigate whether EGFR mutations have effects on PD-L1 expression, we searched TCGA database and found that the mRNA levels of PD-L1 were independent of those of EGFR (Fig. 2A), but correlated with EGFR mutations (Fig. 2B). Flow cytometry was used to detect the expression of PD-L1 on the cell membrane surface. EGF with increasing gradient (25, 50, and 75 ng/mL) induced PD-L1 in H1975 cells in a dose-independent way (Fig. 2C). Furthermore, EGF (50 ng/mL) induced the expression of membrane PD-L1 in H1975 and PC9 cells, but not in H1299 and H460 cells (Fig. 2D). The reason should be that H1975 and PC9 are EGFR-mutated cells, while H1299 and H460 are EGFR wild-type cells 4.

The relationship between MUC3A and EGF-induced PD-L1 in mutated and wild type EGFR cells was then investigated. ShRNAs targeting MUC3A were delivered into the H1975, PC9 and H1299 cells by lentiviruses, and the knockdown efficiency in the stable cell lines was confirmed by immunoblotting (Fig. S2). Flow cytometry results indicated that MUC3A deficiency inhibited EGF-induced PD-L1 expression on the cell membrane surface of H1975 and PC9 cells, but not H1299 cells (Fig. 2E-F). The results of qPCR were consistent. MUC3A deficiency decreased EGF-induced PD-L1 mRNA levels in H1975 and PC9 cells (Fig. 2G). These results indicated that knockdown of MUC3A inhibited EGF-induced PD-L1 expression in EGFR-mutant NSCLC cells, but not in EGFR-wild type cells, suggesting that MUC3A functioned upstream of EGFR.

### MUC3A deficiency suppressed PD-L1 expression via blocking PI3K/Akt and MAPK pathways

**Figure 3:**
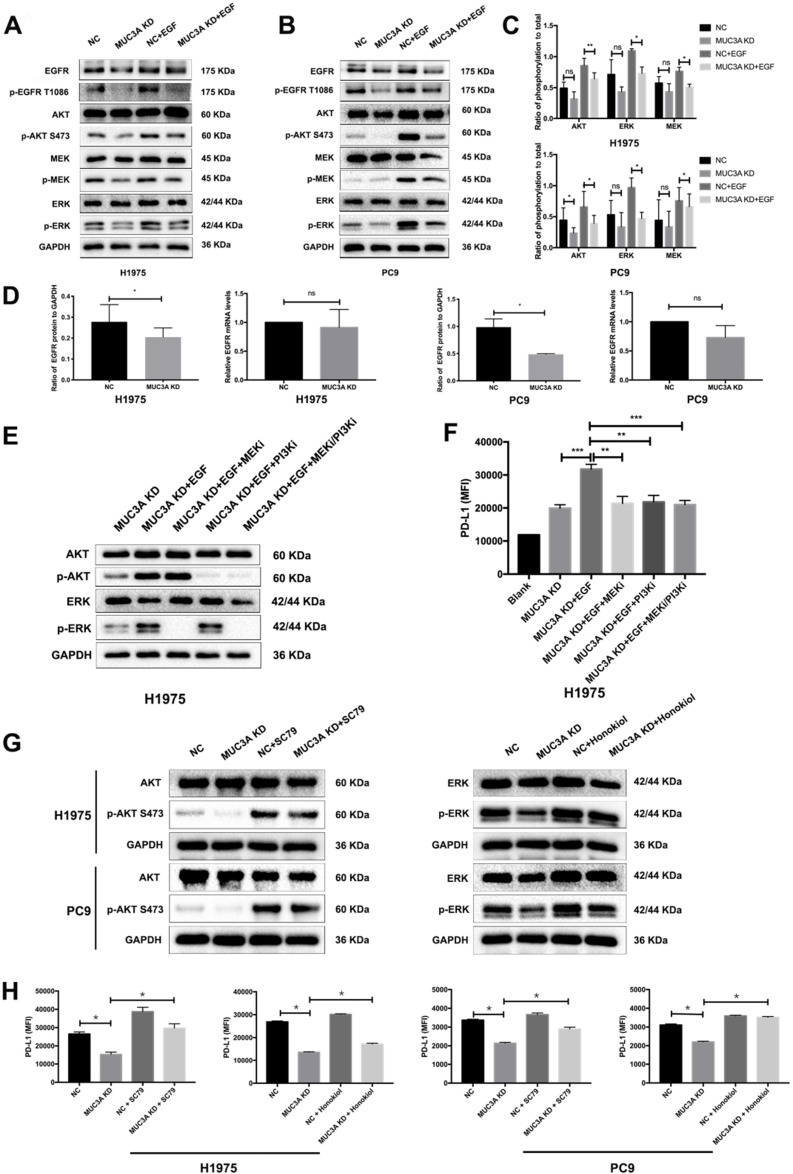
** MUC3A promoted the activity of PI3K/Akt and MAPK pathway. (A)** and** (B)** MUC3A promoted the activity of PI3K/Akt and MAPK pathway, and knockdown of MUC3A decreased EGFR protein levels. Representative immunoblotting of total and phosphorylated EGFR, AKT, MEK and ERK in H1975 and PC9 cells. **(C)** Quantifications of phosphorylated AKT, MEK and ERK**. (D)** MUC3A knockdown had no effect on EGFR mRNA levels, but decreased EGFR protein levels.** (E)** Immunoblotting of PI3K/Akt and MAPK pathway proteins with MEK and PI3K inhibitor treatment for 1 hour.** (F)** Flow cytometry of PD-L1 expression on the surface of H1975 cells treated with EGF, MEK and PI3K inhibitor for 24 hours. **(G)** Immunoblotting of PI3K/Akt and MAPK pathway proteins with AKT and ERK activator treatment for 1 hour. **(H)** Flow cytometry of PD-L1 expression on the surface of H1975 and PC9 cells treated with SC79 or honokiol for 24 hours. Abbreviations are as follows: MFI, mean fluorescence intensity; EGF, 50 ng/mL; MEK inhibitor GSK1120212, 25 nM; PI3K inhibitor GDC-0941, 500 nM; AKT activator SC79, 5 μg/mL; ERK activator honokiol, 20 μM. *, *p* < 0.05; **, *p* < 0.01; ***, *p* < 0.001; ns: not significant. NC: negative control.

Activation of PI3K/Akt and MAPK was reported to be associated with upregulated PD-L1 expression in NSCLC cells 20. One hour after EGF stimulation, reduced PI3K/Akt and MAPK activation was detected in the MUC3A-deficient H1975 and PC9 cells (Fig. 3A-C), which might result from the decreased protein levels of EGFR caused by MUC3A knockdown (Fig. 3A). However, the mRNA levels of EGFR were not affected by the lack of MUC3A (Fig. 3D), suggesting that knockdown of MUC3A downregulated EGFR via reducing its protein translation or stability, instead of mRNA transcription.

To confirm the involvement of PI3K/Akt and MAPK pathways in MUC3A-mediated PD-L1 modulation, specific inhibitors of MEK, GSK1120212 (trametinib), and pan type I PI3Ks, GDC-0941 (pictilisib), were used to treat NSCLC cells (Fig. 3E). MEK and PI3K inhibitors blocked EGF**-**induced PD-L1 expression in MUC3A-deficient H1975 cells (Fig. 3F). Activators of AKT, SC79, and activators of ERK, honokiol, were used to treat NSCLC cells (Fig. 3G). It was observed that both the activators could restore Akt and ERK activation attenuated by MUC3A, and restore the expression of PD-L1 (Fig. 3H). These results indicated that MUC3A knockdown inhibited PD-L1 expression induced by EGF through PI3K/Akt and MAPK pathway.

### Knockdown of MUC3A improved NSCLC sensitivity to TKIs *in vitro*

**Figure 4:**
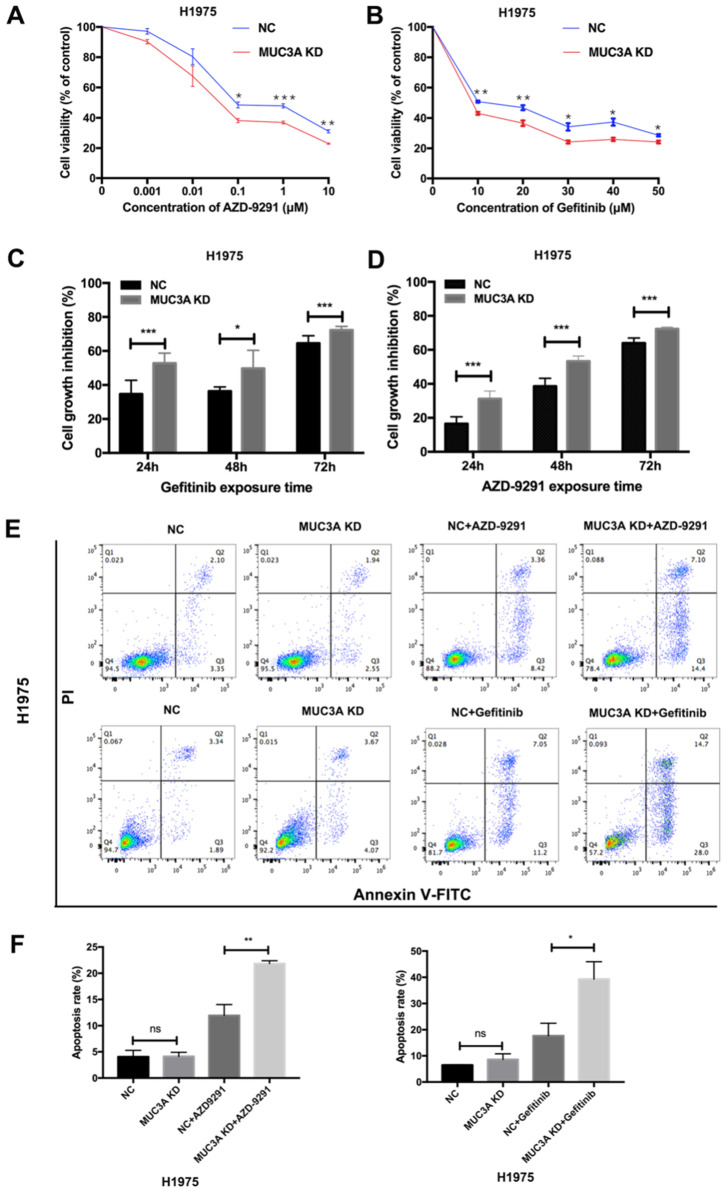
** MUC3A deficiency improved NSCLC sensitivity to TKIs *in vitro*. (A)** and** (B)** MUC3A deficiency potentiated gefitinib and AZD-9291-induced growth inhibition in H1975 cells. **(C)** and** (D)** Cell growth inhibition after exposed to AZD-9291 (0.1 μM) and Gefitinib (10 μM) for 24, 48 and 72 hours.** (E)** and **(F)** MUC3A knockdown increases NSCLC cell apoptosis induced by Gefitinib (10 μM) and AZD-9291 (0.1 μM). *, *p* < 0.05; **, *p* < 0.01; ***, *p* < 0.001; ns: not significant. NC: negative control.

MUC3A deficiency reduced the levels of EGFR proteins, we next explored whether MUC3A affected the *in vitro* drug sensitivity of EGFR-mutant cells to TKIs. Two TKIs were selected, AZD-9291 (sensitive to H1975) and gefitinib (resistant to H1975). The results of cell viability assay indicated that MUC3A deficiency increased the inhibitory effects of TKIs on H1975 cell growth in a dose-dependent manner (Fig. 4A-B). TKIs also markedly inhibited the cell growth of MUC3A-deficient H1975 cells in a time-dependent manner (Fig. 4C-D). Moreover, knockdown of MUC3A increased TKIs-induced cell apoptosis in H1975 cells (Fig. 4E-F). These results suggested that MUC3A deficiency promoted TKIs sensitivity in NSCLC cells *in vitro*.

### MUC3A deficiency improved NSCLC sensitivity to TKIs *in vivo*

**Figure 5:**
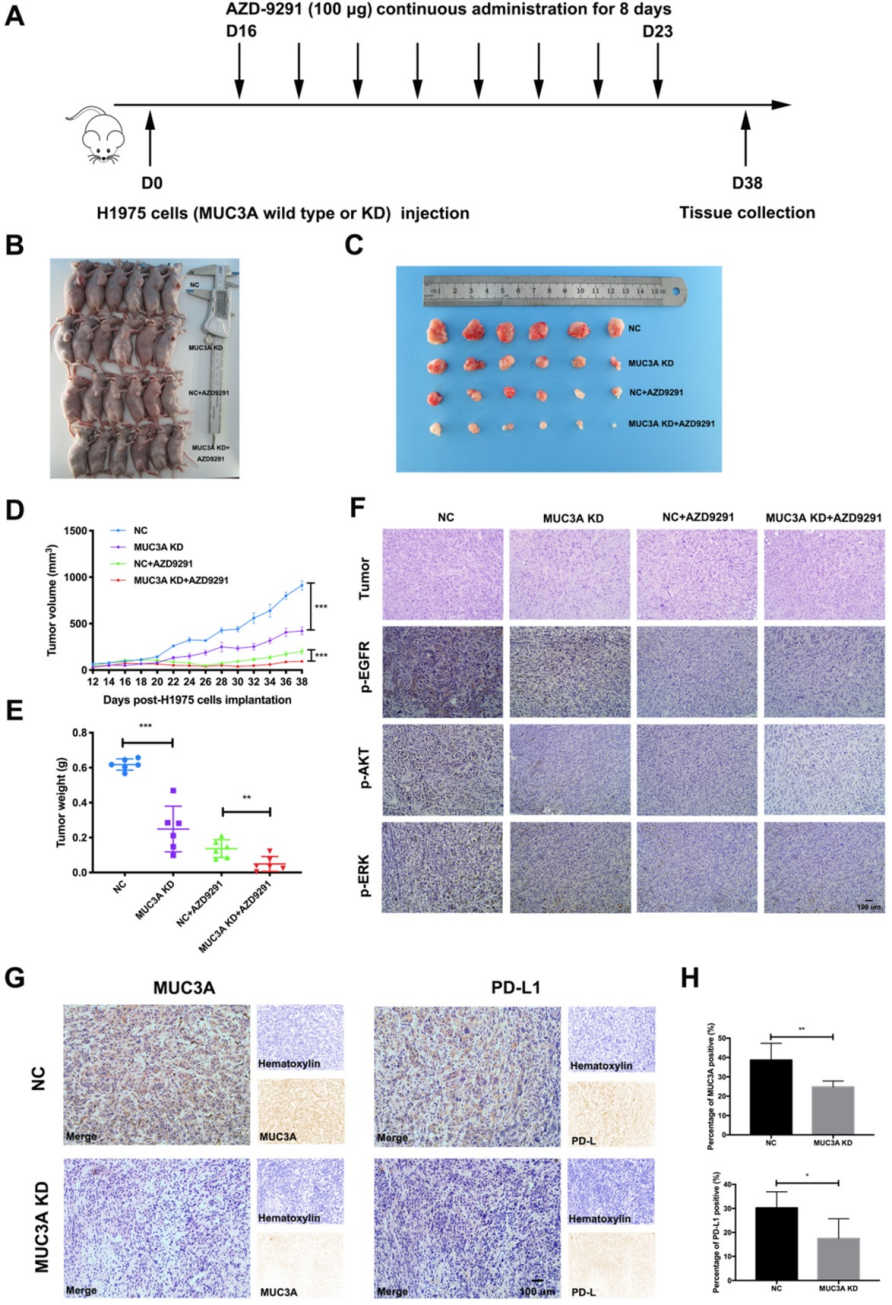
** MUC3A deficiency improved NSCLC sensitivity to TKIs *in vivo*. (A)** The overall scheme of animal experiments. **(B)** Twenty-four nude mice were sacrificed on day 38. **(C)** Gross view of tumor. N = 6. **(D)** Growth curves of tumor volume indicate that the combination therapy significantly inhibits tumor growth *in vivo* compared with single treatment groups. N = 6. **(E)** Tumor weight. N = 6. **(F)** Representative H&E staining images of tumor, and representative IHC images of p-EGFR, p-AKT and p-ERK, scale bar: 100 μm. **(G)** and** (H)** Representative IHC images of MUC3A and PD-L1 in tumor tissues. The MUC3A deficiency group had less MUC3A and PD-L1 expression than that of control. *, *p* < 0.05; **,* p* < 0.01; ***, *p* < 0.001; ns: not significant. NC: negative control.

To investigate the effects of MUC3A on NSCLC cell sensitivity to TKIs *in vivo*, MUC3A-deficient or wild-type H1975 cells were subcutaneously implanted into the BALB/c nude mice, followed by AZD-9291 treatment (100 μg/mouse/day for 8 days, Fig. 5A). MUC3A deficiency significantly suppressed tumor volume, and the combination with AZD-9291 exerted synergistic effect on the reduction of tumor burden (Fig. 5B-E). H&E staining was performed to confirm the tumor tissues (Fig. 5F). Moreover, p-EGFR, p-AKT and p-ERK were decreased in the MUC3A deficient cells after treatment with AZD-9291. IHC of the tumor sections was used to confirm MUC3A knockdown by shRNA lentiviruses, and the xenografts from the mice injected with MUC3A-deficient cells presented significantly less MUC3A and PD-L1 (Fig. 5G-H). These results suggested that MUC3A deficiency impaired tumor growth and promoted sensitivity to TKIs *in vivo*.

## Discussion

**Figure 6:**
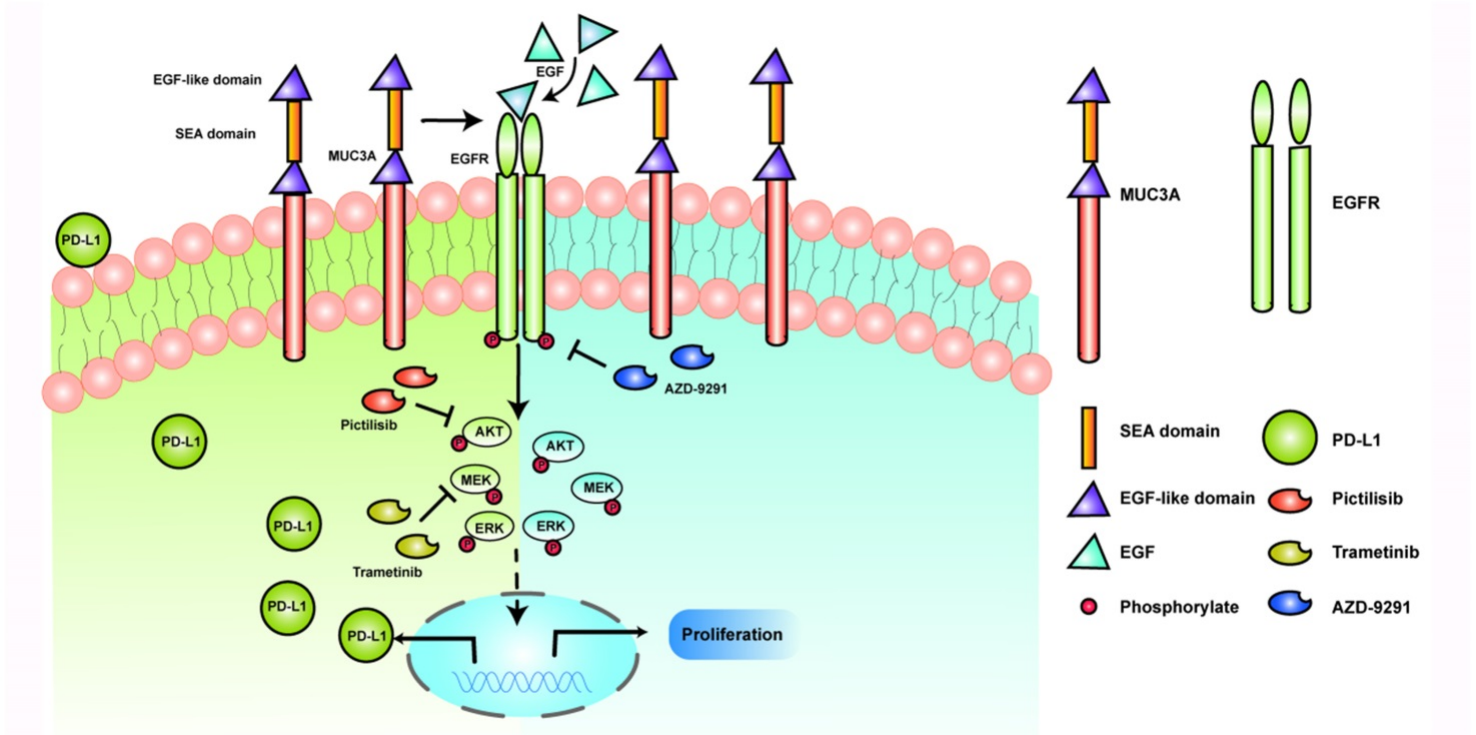
** Schematic model proposed for the role of MUC3A inducing PD-L1 and reducing TKI effects in EGFR-mutant NSCLC.** MUC3A induced PD-L1 via PI3K/AKT and MAPK pathway. MEK and PI3K inhibitors (trametinib and pictilisib) blocked EGF-induced PD-L1 expression. TKIs (AZD-9291) inhibited the cell proliferation.

MUCs have been identified as adverse prognosis markers and attractive therapeutic targets 5. In our study, we identified that MUC3A induced PD-L1 and reduced TKIs effects in EGFR-mutant NSCLC (Fig. 6). MUC3A was reported to be highly expressed in human lung cancer 23, 24. Our tissue microarray showed the same results that MUC3A was highly expressed in non-small cell lung cancer. Moreover, PD-L1 was also upregulated in NSCLC, and positively related with MUC3A. PD-L1 is one of the most important predictors for the efficacy of NSCLC anti-PD-L1 related-immunotherapy 25. Numerous studies suggested that mutant-EGFR signaling induced PD-L1 expression 4, 22, 26, 27. In our study, we revealed that MUC3A altered the levels of PD-L1 via affecting the stability of mutant EGFR protein.

Transmembrane protein MUC3A contains an extracellular cysteine-rich domain with 2 EGF-like motifs 28. Theoretical structure-function relationship analysis of the conserved domains indicated that all of the MUCs of this subfamily could interact with ErbB family member 29. EGFR is a member of the ErbB family and its mutation is one of the most common drivers in NSCLC 30. Compared with wild-type EGFR, EGFR mutation increases both mRNA and protein levels of PD-L1 3, 31. Based on the TCGA database, we found that the expression levels of PD-L1 in EGFR-mutant lung cancer were significantly increased compared with wild-type EGFR one. Previous studies demonstrated that MUC1C induced PD-L1 and immune evasion in TNBC 8. MUC3A shares the same SEA domain with MUC1, suggesting that this domain is critical for its autoproteolysis to impact EGF-induced functions. Upon EGF stimulation, the PD-L1 expression of EGFR mutant cell lines H1975 and PC9 was significantly upregulated, while the PD-L1 expression in EGFR wild-type cell lines H1299 and H460 did not change significantly in our study. After MUC3A knockdown in EGFR-mutant and wild-type NSCLC cells, the expression of PD-L1 was reduced only in EGFR-mutant cells upon EGF stimulation. However, other studies showed that EGFR activation by EGF stimulation upregulated PD-L1 expression in EGFR wild-type BEAS-2B cells 22. There may be several reasons for this discrepancy. One possible reason is the difference of PD-L1 expression baselines. Another reason may be the different protocols of EGF stimulation. In brief, our current results indicated that MUC3A induced PD-L1 in EGFR-mutant NSCLC cells.

Upregulation of PD-L1 was reported to be modulated by activation of MAPK 32, 33 and PI3K/Akt 32, 34, 35, as well as transcriptional factors HIF-1α 36, STAT3 37 and NF-ĸB pathyway 38. In our study, knockdown of MUC3A downregulated phosphorylation of MEK, ERK and AKT. Flow cytometry results showed that MEK and PI3K inhibition reduced the expression of membrane PD-L1. Our results suggested that MUC3A induced PD-L1 through MAPK and PI3K/Akt pathways.

MUC1-mediated protection against EGFR degradation can increase total cellular pools of EGFR over time 39, 40. MUC4 was reported to modulate the expression of EGFR 9. MUC3A has a domain structure similar to MUC4, with N-terminal tandem repeats, EGF-like sequence and a SEA module 5. Our results demonstrated that MUC3A deficiency significantly decreased the protein levels of EGFR, instead of mRNA levels. These studies indicated that MUC3A affected the stability of the EGFR proteins.

Tumors with constitutively active EGFR mutations were reported in up to 40% cases of NSCLC in Asian populations 41. TKIs targeting mutant EGFR reduce the cancer growth successfully, but acquired resistance inevitably occurs 42. Degradation of endogenous mutant EGFR is a common mechanism for the most clinically relevant TKIs-sensitizing 43. Our results showed here that MUC3A deficiency increased TKIs-induced NSCLC cells proliferation inhibition and apoptosis. Our studies further indicated that MUC3A reduced TKIs effects in EGFR-mutant NSCLC via increasing the stability of EGFR proteins. Moreover, i*n vivo* studies collectively supported the conclusion that MUC3A deficiency increased TKIs drug sensitivity and downregulated PD-L1 expression.

Mutant EGFR induces PD-L1 via PI3K/Akt and MAPK pathway. We demonstrated that the transmembrane mucin MUC3A increased EGFR protein stability in EGFR-mutant cell lines. MUC3A can also reduce the effect of TKIs through EGFR modulation in NSCLC. Our studies provided rationale to target MUC3A combining with TKIs in EGFR-mutant lung cancers.

## Supplementary Material

Supplementary figures and tables.

## Figures and Tables

**Table 1:** Correlation between MUC3A and PD-L1 levels in NSCLC patients and their clinicopathologic characteristics.

Clinical pathology	MUC3A^low^PD-L1^low^	MUC3A^high^ PD-L1^low^	MUC3A^low^ PD-L1^high^	MUC3A^high^ PD-L1^high^	N	*p* value
Gender						
Male	21	3	17	10	51	*p*=0.1552
Female	18	2	17	4	41	
Age						
≤ 60	20	0	17	1	38	*p*=0.2710
> 60	19	5	17	13	54	
Tumor size (cm)						
< 4	19	0	23	3	45	*p*=0.5735
≥ 4	16	3	8	7	34	
None	4	2	3	4	13	
Histological grade						
I/I-II	4	0	1	2	7	*p*=0.0577
II	24	2	19	5	50	
II-III/III	9	2	13	7	31	
I-III	2	1	1	0	4	
Clinical Stage						
I	11	1	9	4	25	*p*=0.0917
II	10	1	7	3	21	
III-IV	10	1	8	2	21	
Non	8	2	10	5	25	
Lymph node status						
< 4	11	0	12	2	25	*p* < 0.05
≥ 4	27	4	21	8	60	
Non	1	1	1	4	7	
Carcinoma						
Primary	39	5	34	14	92	*p* < 0.05
Adjacent	86	2	0	0	88	

*P* value represents the probability from ANOVA for tissue PD-L1 and MUC3A levels between variable subgroups.

## Abbreviations

NSCLC: non-small cell lung cancer
TKI: tyrosine kinase inhibitor
MUC: mucin
LUAD: lung adenocarcinoma
EGFR: epidermal growth factor receptor
EGF: epidermal growth factor
TNBCs: triple negative breast cancers
SEA: sperm protein, enterokinase, agrin
IHC: Immunohistochemistry
H&E: hematoxylin and eosin
