# Supplementary material for: MUC3A induces PD-L1 and reduces tyrosine kinase inhibitors effects in EGFR-mutant non-small cell lung cancer
Source: Int J Biol Sci. 2021 Apr 12;17(7):1671–81. doi: 10.7150/ijbs.57964 (PMC8120466; doi:10.7150/ijbs.57964)
Supplement: Supplementary file 1 — Supplementary figures and tables. [file ijbsv17p1671s1.pdf]

Figure S1

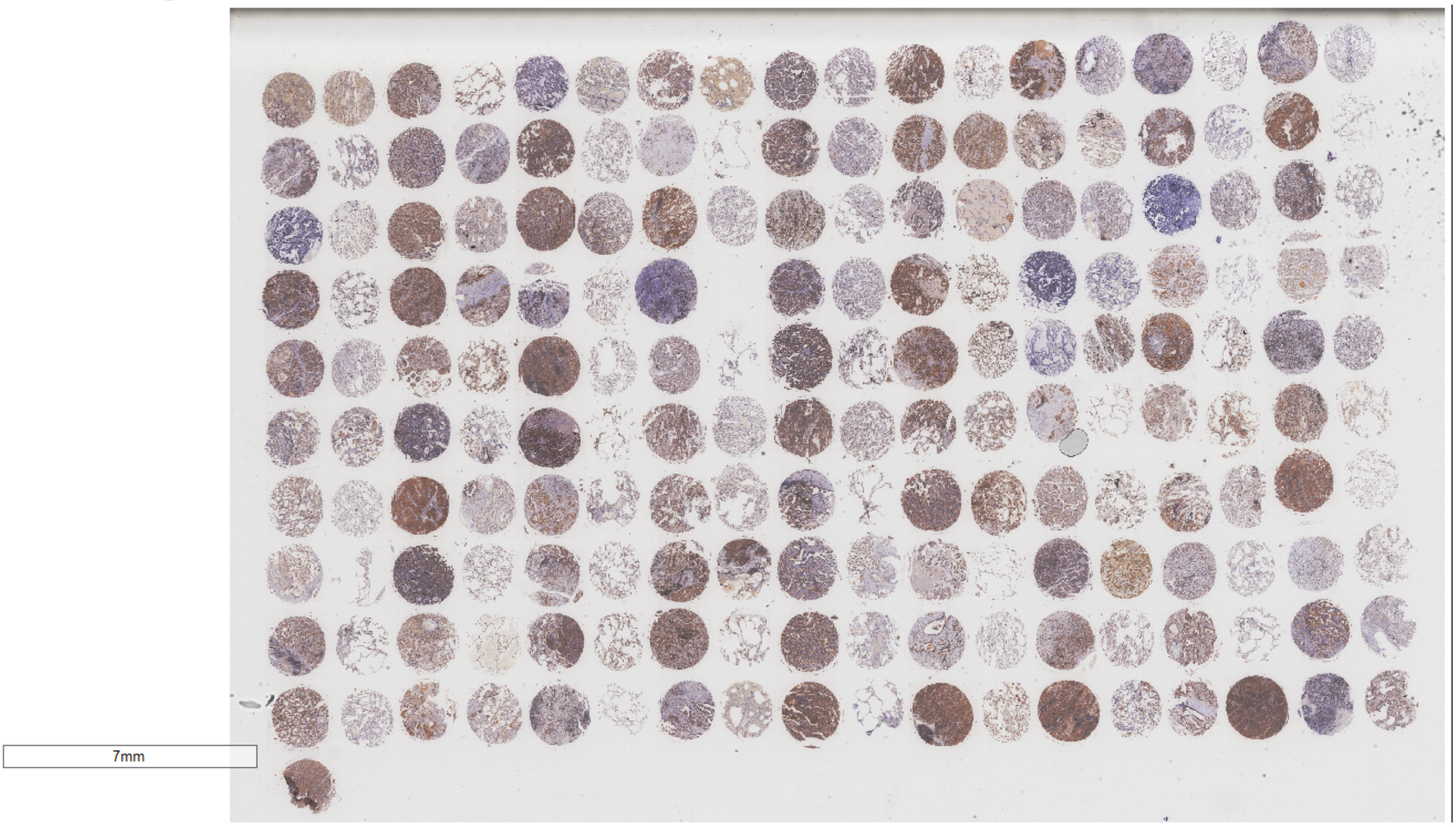

Figure S2

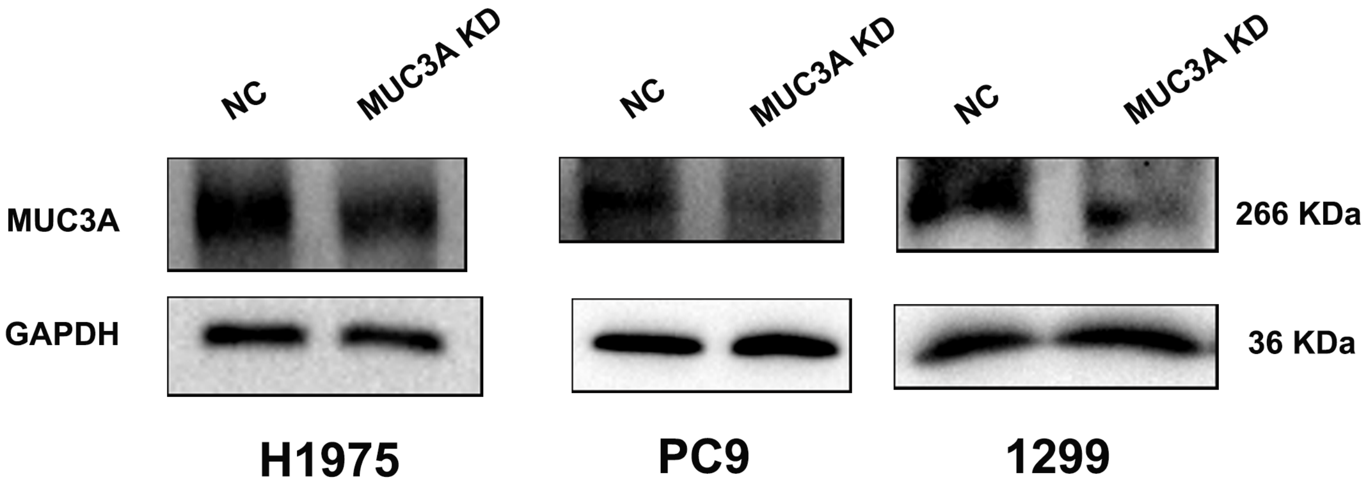
NC
MUC3A KD
NC
MUC3A KD
NC
MUC3A KD
MUC3A
GAPDH
266 KDa
36 KDa
H1975
PC9
1299

Table S1

REAGENT or RESOURCE	SOURCE	IDENTIFIER
MUC3A	abcam	Cat#ab138510
PD-L1	BD biosciences	Cat#557924
EGFR	proteintech	Cat#66455-1-Ig
p-EGFR	Cell Signaling Technology	Cat#2220S
AKT	Cell Signaling Technology	Cat#4691
p-AKT	Cell Signaling Technology	Cat#4060
MEK	Cell Signaling Technology	Cat#11049
p-MEK	Cell Signaling Technology	Cat#9154
ERK	proteintech	Cat#16443-1-AP
p-ERK	Cell Signaling Technology	Cat#4370
GAPDH	proteintech	Cat#10494-1-AP
HRP-conjugated Affinipure Goat Anti-Rabbit IgG(H+L)	proteintech	Cat#SA00001-2
HRP-conjugated Affinipure Goat Anti-Mouse IgG(H+L)	proteintech	Cat#SA00001-1
MEK inhibitor	MedChemExpress	Cat#HY-10999A
PI3K inhibitor	MedChemExpress	Cat#HY-50094
SC79	MedChemExpress	Cat#HY-18749
Honokiol	MedChemExpress	Cat#HY-N0003

Table S2 Correlation between PD-L1 levels in NSCLC patients and their clinicopathologic characteristics

Clinical pathology	PD-L1^{low}	PD-L1^{high}	N	*p* value
Gender				
Male	24	27	51	$p = 0.869$
Female	20	21	41	
Age				
≤ 60	20	18	38	$p = 0.439$
> 60	24	30	54	
Tumor size (cm)				
< 4	19	26	45	$p = 0.229$
≥ 4	19	15	34	
None	6	7	13	
Histological grade				
I/I-II	4	3	7	$p = 0.300$
II	26	24	50	
II-III/III	11	20	31	
I-III	3	1	4	
Clinical Stage				
I	12	13	25	$p = 0.942$
II	11	10	21	
III-IV	11	10	21	
Non	10	15	25	
Lymph node status				
< 4	11	14	25	$p = 0.519$
≥ 4	31	29	60	
Non	2	5	7	
Carcinoma				
Primary	44	48	92	$p < 0.0001$*
Adjacent	88	0	88	

P value represents the probability from a chi-square test for tissue PD-L1 levels between variable subgroups, *$p < 0.05$.
